# Whole-Genome Sequences of Pantoea agglomerans BL3, Pseudomonas fluorescens BL, and Pseudomonas stutzeri CM14, Isolated from Hops (Humulus lupulus)

**DOI:** 10.1128/MRA.00545-19

**Published:** 2019-07-25

**Authors:** Joseph L. Sevigny, Breana Lloyd, Catherine McComish, Alexis Ramsey, Lori Koziol

**Affiliations:** a Hubbard Center for Genome Studies, University of New Hampshire, Durham, New Hampshire, USA; b Department of Biology, New England College, Henniker, New Hampshire, USA; University of Arizona

## Abstract

Pseudomonas stutzeri CM14, Pseudomonas fluorescens BL, and Pantoea agglomerans BL3 were isolated from Humulus lupulus cones. Here, we present the draft genome sequences of three bacteria that have been associated with hop plants.

## ANNOUNCEMENT

Humulus lupulus, also known as hops, is one of the four main ingredients used to produce beer (1). This study sequenced the genomes of three bacteria isolated from hops. Since whole hop cones may be added to the brewing process after the boil, bacteria associated with hop cones may play a role in beer production. The genome sequencing of these bacteria and the shared metabolic pathways of carbohydrates may be used to inform brewers of the potential flavor profile of beers.

Pantoea agglomerans BL3 and Pseudomonas stutzeri CM14 were isolated from a hop cone from a residential New Hampshire location in August 2017. The hop cone was placed in phosphate-buffered saline (PBS), vortexed, and plated onto nutrient agar plates. Pseudomonas fluorescens BL was isolated from another hop cone from the same New Hampshire location in August 2018; this cone was vortexed in PBS and plated onto violet-red glucose agar plates. A single colony was selected and grown overnight in liquid lysogeny broth (LB), and genomic DNA was isolated using the Wizard SV genomic DNA purification system (Promega, Madison, WI). The whole-genome sequencing (WGS) of the bacterium was performed at the Hubbard Center for Genome Studies (University of New Hampshire, Durham, NH, USA). WGS libraries were prepared following the Kapa Biosystems HyperPlus kit (KR1145 -v3.16). Sequencing was completed on an Illumina HiSeq 2500 instrument for 518 cycles to produce 250-bp paired-end reads. Raw sequencing data were demultiplexed using the Illumina bcl2fastq conversion software v1.8.4. TruSeq adapters and low-quality bases were trimmed using Trimmomatic v0.36 under default settings (2). An initial assembly was constructed from the trimmed reads using SPAdes v3.11.0 (3). Assembly contiguity and completeness were assessed using QUAST v4.6.0 and BUSCO v3.0.0, respectively (4, 5). Contaminant sequences (those that originated from a nontarget organism) were identified and removed from the assembly following the blobtoolkit pipeline (6). Contiguous sequences (contigs) were selectively removed from the assembly if the average read coverage was below 5×. In each case, these low-coverage contigs were also identified as a different taxonomic group with BLAST. The retained contigs best matched to the expected target genera and have greater than 100× coverage. Default parameters were used for all software, unless otherwise specified.

The draft genome assemblies of the three hop bacteria, Pantoea agglomerans BL3, Pseudomonas stutzeri CM14, and Pseudomonas fluorescens BL, are described in Table 1. The microbes were identified using whole-genome BLAST, and the NCBI confirms the identity using average nucleotide identity (ANI). An ANI test compares the submitted genome sequence against the genomes of the type strains and proxytype strains that are already in GenBank (7). The assembled bacterial genomes were annotated via the NCBI Prokaryotic Genome Annotation Pipeline (PGAP) (8). Pantoea agglomerans BL3 has been found in the rhizosphere and as an endophyte in plant tissues and is generally considered to be beneficial to plant growth; however, *P. agglomerans* has caused occupational disease in people working with hops (9, 10). Both Pseudomonas fluorescens and Pseudomonas stutzeri are known plant-colonizing bacteria (11, 12). This is the first report to identify and sequence these bacteria isolated from *Humulus lupulus* cones.

**TABLE 1:** Characteristics and accession numbers of bacterial genomes isolated from hops

Isolate	Bacterial species	Genome size (bp)	Estimated coverage (×)	No. of contigs	*N*_50_ (kb)	Total no. of genes	No. of rRNAs	No. of tRNAs	G+C content (%)	Genome assembly accession no.
BL3	Pantoea agglomerans	5,046,415	170	22	420	4,708	15	68	55.2	GCA_004793995
CM14	Pseudomonas stutzeri	4,677,845	188	55	209	4,368	3	54	63.9	GCA_004793985
BL	Pseudomonas fluorescens	5,882,262	1,340	62	374	5,355	6	59	60.5	GCA_004794015

### Data availability.

This whole-genome shotgun project was deposited at DDBJ/ENA/GenBank under the accession numbers GCA_004793995, GCA_004793985, and GCA_004794015. Raw sequencing reads are available in the NCBI Sequence Read Archive under the accession numbers SRR8872446, SRR8872447, and SRR8872448 for P. stutzeri, *P. agglomerans*, and P. fluorescens, respectively.
